# Rewiring the seizing brain: stem cell grafts as neuromodulatory architects in epilepsy therapy

**DOI:** 10.3389/fneur.2025.1596484

**Published:** 2025-08-22

**Authors:** Zijie Wang, Yanan Ma, Xiqi Hu, Ying Xia

**Affiliations:** Department of Neurosurgery, Haikou Hospital Affiliated with Xiangya Medical College, Central South University, Haikou, China

**Keywords:** stem cell transplantation, refractory epilepsy, modulation of oxidative stress, inhibitory interneurons, clinical translation

## Abstract

As an emerging therapeutic strategy, stem cell transplantation has demonstrated promising potential in the management of refractory epilepsy. Epilepsy, a prevalent neurological disorder characterized by recurrent seizures, affects approximately one-third of patients worldwide who exhibit resistance to existing antiepileptic drugs (AEDs). Consequently, exploring novel treatment modalities is imperative. Recent studies have indicated that stem cell transplantation improves neurological function in epilepsy through multiple mechanisms. Research has revealed that transplanted stem cells mitigate seizure-induced neuronal damage by replacing dead or dysfunctional neurons, secreting beneficial neurotrophic factors (e.g., brain-derived neurotrophic factor, BDNF), and releasing anti-inflammatory cytokines. Preclinical studies and early-phase clinical trials have shown that stem cell transplantation significantly reduces seizure frequency and enhances patients’ quality of life. However, current research is limited by small sample sizes and short-term follow-ups, necessitating further validation of long-term efficacy. Despite its therapeutic promise, stem cell transplantation faces critical challenges. First, technical details such as the cell source, processing, method of transplantation, and timing have yet to be standardized, leading to potential variability in efficacy and safety across different stem cell types. Second, complications like immune rejection and tumorigenesis pose significant safety risks. Future research should focus on optimizing stem cell selection and processing, designing robust clinical trials to evaluate long-term safety and efficacy, exploring combinatorial approaches with existing therapies, and developing advanced biomaterials to enhance transplantation success. Additionally, monitoring post-transplant cell survival and functionality, along with identifying epilepsy-specific biomarkers, will be crucial to refining the precision and safety of stem cell-based therapies.

## 1 Introduction

Epilepsy represents a prevalent neurological disorder characterized by recurrent seizures arising from abnormal hypersynchronous neuronal electrical activity in the brain (1). Affecting over 65 million individuals worldwide, epilepsy constitutes a significant global public health challenge (2). The clinical manifestations of epileptic seizures encompass transient paroxysmal signs and symptoms resulting from excessive neuronal discharges, with classification into focal onset seizures (originating within discrete neural networks) and generalized seizures (involving bilateral distributed networks), the latter frequently presenting with impaired consciousness and bilateral motor manifestations (2). The etiological spectrum of epilepsy is remarkably diverse, encompassing genetic predisposition, structural brain injuries (e.g., traumatic brain injury from accidents or falls), central nervous system infections (including meningitis and encephalitis), neoplastic lesions, metabolic disturbances (such as hypoglycemia or hypocalcemia), and neurodegenerative conditions (3, 4). Notably, a substantial proportion of cases (approximately 30–40%) remain classified as idiopathic epilepsy with undetermined etiology despite extensive diagnostic evaluation (5). Status epilepticus (SE), a severe and emergent condition within epilepsy, is operationally defined as a seizure persisting beyond 5 min or two or more discrete seizures without full recovery of consciousness between them. According to the International League Against Epilepsy (ILAE) 2015 definition, SE is a condition resulting either from the failure of the mechanisms responsible for seizure termination or from the initiation of mechanisms that lead to abnormally prolonged seizures. For generalized convulsive status epilepticus (GCSE), the operational time points are defined as t1 = 5 min (the time at which a seizure is considered prolonged and unlikely to stop spontaneously, thus warranting treatment) and t2 = 30 min (the time at which there is a significant risk of long-term complications) (6). Status epilepticus (SE) manifests in various forms. Common types include: (1) Generalized Convulsive Status Epilepticus (GCSE): Characterized by paroxysmal or sustained rhythmic tonic, clonic, or tonic–clonic muscle activity, impaired consciousness during seizures, and lack of complete consciousness recovery *between* seizures. It is frequently accompanied by severe autonomic symptoms including hyperpyrexia, tachycardia, respiratory abnormalities, blood pressure fluctuations, pupillary changes, and pathological reflexes. (2) Non-Convulsive Status Epilepticus (NCSE): Absence Status Epilepticus (ASE): Predominantly affects children, presenting with abrupt, relatively mild impairment of consciousness manifesting as lethargy, confusion, reduced voluntary movement, and slowed speech. Complex Partial Status Epilepticus (CPSE): Exhibits diverse levels of impaired consciousness and EEG abnormalities that are periodic and prolonged. Patients may remain in a twilight state for extended periods, accompanied by unresponsiveness and automatisms. (3) Simple Partial Status Epilepticus (SPSE): Consciousness is largely preserved. Patients experience continuous focal sensory or motor seizures. Transient post-ictal hemiparesis (Todd’s paralysis) may occur in the affected region following seizure cessation (6, 7). Failure to effectively control status epilepticus (SE) may lead to progression into refractory status epilepticus (RSE), defined as persistence of seizures despite adequate treatment with first-line (benzodiazepines) and second-line (e.g., levetiracetam, phenytoin, valproate sodium, or phenobarbital) antiseizure medications. This may further progress to super-refractory status epilepticus (SRSE), characterized by ongoing or recurrent seizures 24 h or more after the initiation of anesthetic therapy, or during its weaning or withdrawal phase. Furthermore, SE is closely associated with the development of chronic epilepsy (8). Repeated or prolonged SE episodes can accelerate the process of epileptogenesis, altering brain neural circuitry and function. This leads to recurrent seizures that are often more difficult to control.

The underlying mechanisms involve multiple aspects, including sustained activation of neuroinflammation, neuronal injury and death, disruption of neurotransmitter systems, and aberrant changes in brain plasticity. For instance, the massive release of pro-inflammatory cytokines during SE not only exacerbates acute neuronal damage but may also chronically alter gene expression and function in neural cells. This impacts the remodeling and stability of neural circuits, thereby promoting epileptogenesis. Concurrently, seizure-induced neuronal death and abnormal neurogenesis can contribute to the formation of aberrant synaptic connections, establishing a structural basis for persistent seizure activity (9, 10).

The pathophysiological mechanisms underlying epileptogenesis involve complex interactions between neuronal hyperexcitability and impaired inhibition. Emerging evidence implicates multiple interconnected biological processes, including substantial loss of specific interneuron subpopulations (particularly somatostatin- and parvalbumin-expressing GABAergic cells), astrocytic dysfunction in glutamate homeostasis, and dysregulation of synaptic plasticity (11, 12). Among these mechanisms, the loss or functional impairment of GABAergic inhibitory interneurons is recognized as one of the core mechanisms underlying hyperexcitability within epileptic networks. These neurons regulate the firing frequency of excitatory neurons through GABA release. Their deficiency disrupts the brain’s excitation-inhibition (E/I) balance, directly triggering or amplifying seizure activity. For example, in patients with temporal lobe epilepsy (TLE), hippocampal tissue exhibits neuronal loss, altered immunohistochemical profiles, and changes in electrophysiological properties. Specifically, in medial temporal lobe epilepsy (mTLE), there is a loss of neurons immunoreactive for neuropeptide Y (NPY), somatostatin (SST), and substance P (SP) within the subgranular polymorphic zone of the dentate gyrus. Furthermore, the mTLE group demonstrates significantly reduced overall hippocampal neuronal density compared to other groups, accompanied by electrophysiological evidence of diminished inhibitory function and enhanced excitability. These pathological alterations are strongly implicated in the pathogenesis of epilepsy (11, 13).

Recent studies have highlighted the critical role of oxidative stress pathways, with ferroptosis—an iron-dependent form of regulated cell death characterized by mitochondrial dysfunction and lipid peroxidation—being implicated in temporal lobe epilepsy pathogenesis (14–16). During epileptic seizures, excessive neuronal firing leads to a surge in oxygen consumption and disruption of mitochondrial respiratory chain function, resulting in massive overproduction of reactive oxygen species (ROS). Concurrently, the activity of the antioxidant defense system – including enzymes such as superoxide dismutase (SOD) and glutathione – is suppressed. This creates an imbalance between oxidative stress and antioxidant capacity. Excess ROS not only directly damage neuronal lipids, proteins, and DNA, but also compromise the integrity of the blood–brain barrier, promote the release of pro-inflammatory cytokines, and further exacerbate mitochondrial dysfunction. This cascade establishes a self-perpetuating vicious cycle of “oxidative stress → neuronal damage → epileptic seizures” (15, 16). Targeting this mechanism, stem cells, particularly mesenchymal stem cells (MSCs), can exert protective effects by mitigating cellular oxidative damage and aberrant mitochondrial function. This is achieved through the secretion of antioxidant factors (such as superoxide dismutase), miRNAs within exosomes, and modulation of the Nrf2-HO-1 antioxidant pathway, thereby promoting neuronal survival (17, 18). Neuroinflammation also plays a significant role in both the initiation and progression of epilepsy. Seizures or brain injury can activate microglia and astrocytes, leading to the release of pro-inflammatory cytokines (such as IL-1β, TNF-*α*, and IL-6) and chemokines. These inflammatory mediators further enhance neuronal excitability and disrupt synaptic homeostasis. For instance, IL-1β enhances neuronal calcium influx by activating NMDA receptors, while TNF-*α* regulates blood–brain barrier permeability and promotes aberrant neurogenesis. This chronic inflammatory microenvironment not only exacerbates acute seizures but also contributes to the chronic process of epilepsy (i.e., epileptogenesis) (19). Moreover, perturbations in ionic homeostasis (particularly involving sodium, potassium, and calcium channels) and genetic mutations affecting voltage-gated or ligand-gated ion channels have been identified as key contributors to neuronal hyperexcitability (20, 21).

Despite the availability of numerous antiepileptic drugs (AEDs) targeting various molecular pathways, approximately 30% of patients develop drug-resistant epilepsy with inadequate seizure control. Current pharmacotherapeutic limitations include significant adverse effects (e.g., cognitive impairment, hepatotoxicity, and metabolic disturbances), narrow therapeutic indices, and the emergence of pharmacoresistance necessitating complex polypharmacy regimens (22). While surgical interventions (e.g., temporal lobectomy or laser ablation) may benefit select patients with focal epileptogenic zones, inherent risks of neurological deficits and variable efficacy underscore the need for alternative therapeutic strategies (23). Recent advances in regenerative medicine have positioned stem cell therapy as a promising investigational approach for refractory epilepsy. Pluripotent and multipotent stem cells possess the remarkable capacity for self-renewal and multilineage differentiation, offering the potential for neural circuit reconstruction through multiple mechanistic pathways (24, 25). Preclinical studies have suggested that stem cell transplantation ameliorates epilepsy through multiple mechanisms: (1) Differentiation into inhibitory interneurons: Transplanted stem cells can differentiate into pallial medial ganglionic eminence (MGE)-derived GABAergic interneurons, restoring inhibitory tone and suppressing seizure activity (26–29); (2) Secretion of neurotrophic factors: Stem cells release neurotrophic factors such as brain-derived neurotrophic factor (BDNF) and glial cell line-derived neurotrophic factor (GDNF), which enhance neuronal survival, maturation, and synaptic plasticity (30–32); (3) Anti-inflammatory modulation: Stem cells mitigate neuroinflammation by secreting cytoprotective, immunomodulatory, and anti-inflammatory factors (33). Stem cells also attenuate neuroinflammatory responses by improving mitochondrial autophagy (34). For example, mesenchymal stem cells (MSCs) attenuate post-epileptic brain injury by upregulating anti-inflammatory cytokines and downregulating pro-inflammatory mediators (19, 35, 36). Despite these promising preclinical findings, critical challenges remain in clinical translation. Key unresolved issues include optimal cell source selection (embryonic vs. induced pluripotent vs. adult stem cells), the method of transplantation (intraparenchymal vs. intravenous delivery), long-term graft survival/functional integration, and the risk of tumorigenicity or aberrant network formation. Moreover, standardization of protocols for cell dosing, timing of intervention relative to epileptogenesis phases, and development of reliable biomarkers for treatment monitoring require systematic investigation. Future research directions should emphasize multimodal approaches combining stem cell therapy with optogenetic modulation or biomaterial scaffolds to enhance graft-host integration. Large-scale randomized controlled trials (RCTs) using standardized seizure outcome measures (e.g., ILAE classification) and advanced neuroimaging modalities (functional MRI, PET) are imperative to establish clinical efficacy. Concurrent basic science investigations must elucidate molecular mechanisms of stem cell-mediated epileptogenesis suppression through single-cell transcriptomics, *in vivo* calcium imaging, and opto−/chemogenetic manipulation of grafted cells. The ultimate therapeutic paradigm will likely require personalized cell therapy regimens tailored to individual epileptogenic network pathologies and genetic profiles.

## 2 Classification of stem cells

Different types of stem cells perform distinct biological functions. To better understand their potential and limitations in biological research and medical applications, thereby enabling more targeted therapeutic strategies, the specific classifications of most stem cells are summarized below.

### 2.1 Classification by origin

#### 2.1.1 Embryonic stem cells

Source: Derived from the inner cell mass of early-stage embryos.

Characteristics: Pluripotent, capable of differentiating into all cell types within the embryo. Although clinical use of ESCs faces scientific, ethical, and legal challenges, clinical trials utilizing human ESCs (hESCs) for therapy have commenced, with protocols established to differentiate hESCs into specialized cell types suitable for transplantation. Scientifically, precisely controlling the differentiation of ESCs into the desired neural cell types remains a complex task. The process needs to be highly regulated to ensure the production of pure populations of functional cells (37, 38). Once the limitations of ESCs are fully overcome, they may become the optimal source of stem cells.

#### 2.1.2 Adult stem cells

Source: Isolated from developed tissues, such as bone marrow, adipose tissue, nervous tissue, and skin.

Characteristics: Exhibit multipotency within their tissue of origin, typically differentiating into lineage-specific cell types. Examples include bone marrow MSCs (BMSCs), neural stem cells (NSCs), and dental pulp stem cells (DPSCs) (39, 40).

#### 2.1.3 Induced pluripotent stem cells

Source: Generated by reprogramming somatic cells (e.g., adult fibroblasts) via genetic or epigenetic modifications to regain pluripotency (41).

Characteristics: iPSCs closely resemble ESCs in their differentiation capacity, enabling generation of cells from all three embryonic germ layers. They also possess robust self-renewal capabilities. A key advantage lies in their autologous origin, minimizing the risk of immune rejection. Additionally, iPSCs can be derived from diverse donor populations (healthy or diseased), circumventing ethical controversies associated with ESCs and broadening their clinical applicability (42–44). iPSCs have emerged as a revolutionary resource in the quest for effective stem cell therapies for epilepsy. Generated by reprogramming somatic cells, such as adult fibroblasts, through genetic or epigenetic modifications to regain pluripotency, iPSCs closely mimic ESCs in their differentiation capacity. The robust self—renewal capabilities of iPSCs further enhance their potential in epilepsy treatment. This characteristic enables the generation of large quantities of cells for transplantation, ensuring an adequate supply for clinical applications. Moreover, a key advantage of iPSCs lies in their autologous origin (45). Since iPSCs can be derived from the patient’s own cells, so the risk of immune rejection is significantly minimized (46). This is a major breakthrough compared to traditional transplantation therapies, as immune-related complications often pose significant challenges in long-term treatment success.

### 2.2 Classification by differentiation potential

#### 2.2.1 Totipotent stem cells

TSCs represent the most potent stem cell type, capable of generating all embryonic and extraembryonic cell lineages (e.g., placenta), thereby supporting the development of a complete organism (47). In the context of epilepsy, this unparalleled potency could, in theory, offer novel therapeutic strategies. However, their use raises significant ethical concerns.

#### 2.2.2 Pluripotent stem cells

PSCs can differentiate into derivatives of all three germ layers (endoderm, mesoderm, ectoderm) but lack the ability to form extraembryonic tissues (48). These cells have been adapted to *in vitro* culture systems distinct from their native developmental niches, facilitating their accessibility and utility in research and therapy (49).

#### 2.2.3 Unipotent stem cells

USCs exhibit the most restricted differentiation potential and are committed to a single cell lineage (e.g., muscle satellite cells) (50). They maintain long-term self-renewal within specific tissues and differentiate to repair localized damage. Their limited ethical controversy, high safety profile, and reduced tumorigenic risk compared to PSCs make them valuable tools in regenerative medicine.

### 2.3 Classification by tissue specificity

#### 2.3.1 Hematopoietic stem cells

HSCs are a class of adult stem cells capable of self-renewal and differentiation into all blood cell lineages and immune cells. They serve as the foundation for the maintenance and regeneration of the hematopoietic and immune systems (51). Clinically, HSCs have been extensively utilized in the treatment of hematologic and immunologic disorders (52, 53). Despite challenges such as donor matching, transplantation-associated complications, and insufficient cell yield, advances in *in vitro* expansion, gene editing, and immune tolerance technologies are expected to broaden their clinical applicability. In the context of epilepsy, emerging research suggests potential roles for HSCs that extend beyond their traditional hematologic and immunologic applications. There is growing evidence of a complex interplay between the immune system and epilepsy (54).

#### 2.3.2 Neural stem cells

NSCs possess the ability to differentiate into diverse cell types of the central nervous system (CNS), including neurons, astrocytes, and oligodendrocytes (55). Their differentiation potential positions NSCs as transformative agents for CNS disease therapies (56). A recent study has highlighted the therapeutic relevance of NSC-derived secretory products, which contribute to host cell survival, neuroplasticity, and neuroimmune modulation (57). In epilepsy, where there is often neuronal damage and impaired neuroplasticity, the secretory products of NSCs can play a crucial role. The paracrine mechanisms of NSCs are considered pivotal for their interaction with neural tissues, as they endogenously generate a multifaceted secretome consisting of growth factors, cytokines, chemokines, morphogens, microRNAs (miRNAs), and other bioactive molecules. For instance, miRNAs within the NSC secretome can regulate gene expression in neighboring cells, influencing processes such as cell survival, differentiation, and synaptic plasticity (58, 59). These properties underscore the immense potential of NSCs in future neurologic disease interventions. As research continues to uncover the complex interplay between NSCs and the epileptic brain, further optimization of NSC-based therapies, including the manipulation of their secretory profiles and differentiation pathways, holds great promise for effectively treating epilepsy and improving the quality of life for patients suffering from this debilitating disorder.

#### 2.3.3 Mesenchymal stem cells

MSCs exhibit multipotent differentiation into mesoderm-derived lineages, such as osteocytes, chondrocytes, adipocytes, myocytes, and bone marrow stromal cells (60, 61). In the context of epilepsy, this multipotency could potentially be harnessed in novel ways. Although the primary goal in epilepsy treatment is often to correct neural dysfunctions, MSCs’ ability to differentiate into stromal cells might contribute to the creation of a more supportive microenvironment in the epileptic brain. For example, bone marrow stromal cell-like derivatives from MSCs could help in remodeling the extracellular matrix, which may be disrupted in epilepsy due to chronic seizures and associated inflammation. This remodeling could, in turn, enhance the survival and function of existing neural cells. Epilepsy is frequently associated with neuroinflammation, which can exacerbate seizure activity and contribute to neuronal damage. MSCs, with their immunomodulatory properties, could play a crucial role in mitigating this inflammation. MSCs are characterized by low immunogenicity, enabling them to suppress excessive immune responses and modulate the local immune microenvironment via secretion of cytokines and bioactive molecules, thereby attenuating inflammatory cascades (62). Preclinical studies have demonstrated that MSCs improve outcomes in animal models of neurologic disorders, including multiple sclerosis, stroke, Alzheimer’s disease, and traumatic brain injury (63). Bone marrow-derived and other tissue-specific MSCs have demonstrated favorable safety profiles in human applications (64, 65). Their versatility in administration routes—such as intravenous, intra-arterial, intraperitoneal, intrathecal, and intranasal delivery—enhances their therapeutic adaptability (66, 67). For instance, intranasal delivery of MSCs could potentially bypass the blood–brain barrier, allowing the cells to directly reach the brain tissue in a more efficient manner. This could be particularly advantageous as the blood–brain barrier may be compromised in epilepsy, but a direct and targeted method of delivery could still optimize the therapeutic effect of MSCs. MSCs are relatively accessible, with human adipose tissue serving as a robust source (68). Additionally, dental-derived MSCs can be isolated from various dental tissues (69), and OM-MSCs located in the nasal cavity have emerged as a viable source of neural stem cells (70). The ease of procurement and long-term viability of OE-MSCs render them particularly suitable for neurologic disease modeling and therapeutic development (71–74). As research progresses, further understanding of the optimal source, differentiation potential, and delivery methods of MSCs will be essential to fully realizing their potential in revolutionizing epilepsy treatment and improving the quality of life for patients suffering from this challenging neurological disorder.

## 3 Stem cell therapy for epilepsy

**Figure 1:**
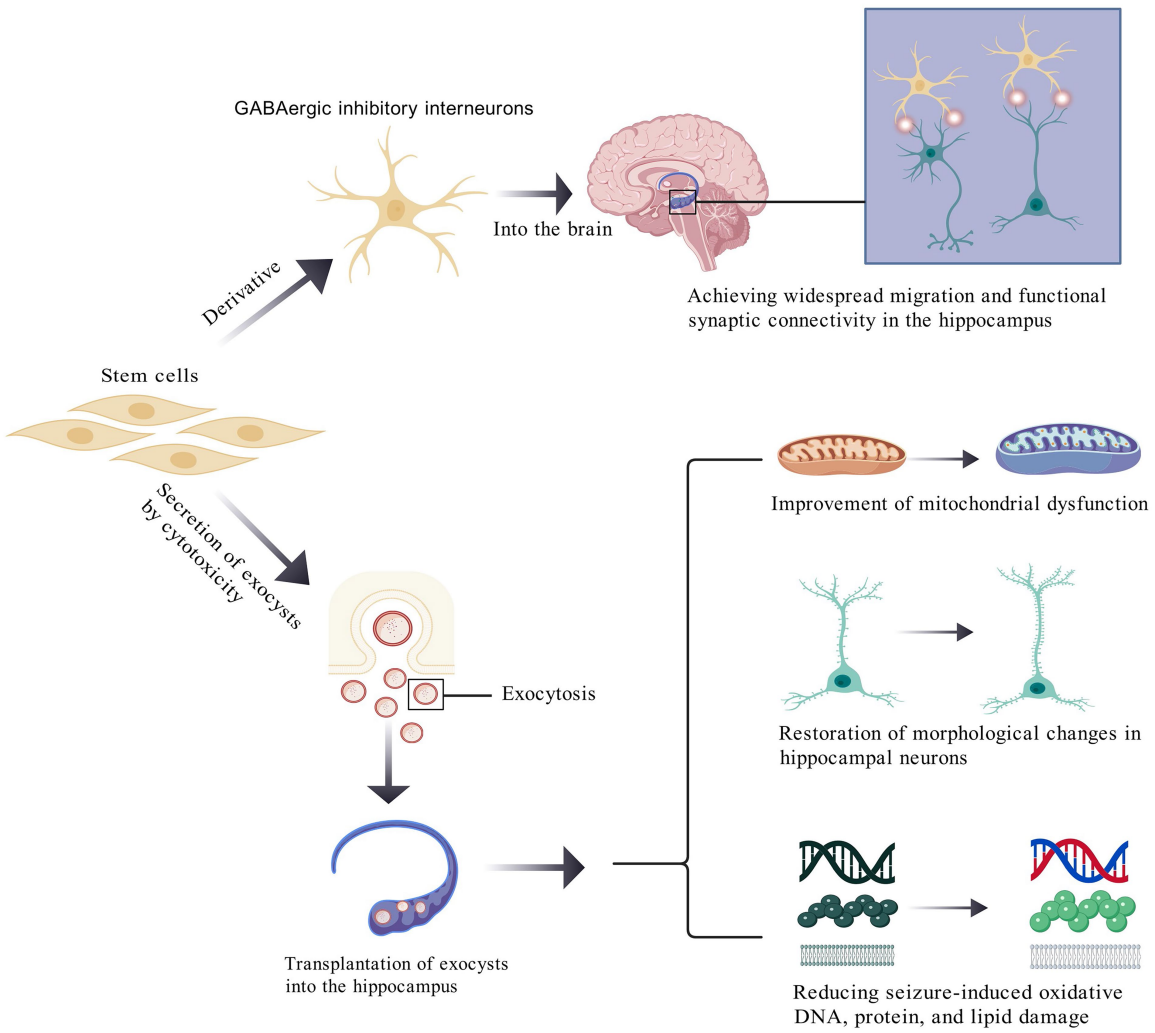
Partial mechanisms of stem cell therapy in epilepsy.

Stem cells injected into the body can alleviate epileptic seizures by differentiating into interneurons (75–79), while also secreting neurotrophic factors to promote neuroprotection and neuroregeneration, thereby improving neural network function (80, 81). Additionally, stem cells may have therapeutic effects by reducing neuroinflammation and modulating immune responses (62, 82–84). Notably, stem cell therapy has demonstrated efficacy in ameliorating spinal muscular atrophy with progressive myoclonic epilepsy (SMA-PME) caused by enzymatic deficiencies (85). Key mechanisms of stem cell therapy in epilepsy are shown in Figure 1.

A key pathophysiological mechanism of epilepsy may involve the loss of inhibitory interneurons. First, stem cell-derived GABAergic inhibitory interneurons can extensively migrate within the hippocampus, functionally integrating into synaptic networks, and releasing inhibitory neurotransmitters to alleviate seizure activity. Second, stem cells release extracellular vesicles (EVs) via exocytosis, which mitigate seizure-induced mitochondrial dysfunction and restore hippocampal neuronal morphology. Post-epilepsy, pyramidal neurons in the hippocampus exhibit shortened total dendritic length, reduced spine density, and altered dendritic morphology. EVs reverse these pathological changes and ameliorate oxidative damage to DNA, proteins, and lipids within hippocampal neurons.

### 3.1 Stem cell differentiation into inhibitory interneurons

Although the precise mechanisms underlying epilepsy remain unclear, the dysfunction or loss of GABAergic inhibitory interneurons is a widely proposed hypothesis. Early studies suggested a link between hippocampal interneuron loss and epilepsy (86). Recent evidence has further indicated that impaired inhibitory interneuron function within hippocampal circuits contributes to seizure generation (87). Therapeutically, Cunningham *et al*. demonstrated that human pluripotent stem cell (PSC)-derived mature GABAergic interneurons (mGINs) extensively migrate and functionally integrate into the brains of post-seizure mice, releasing GABA to suppress synaptic excitation via presynaptic inhibition of host hippocampal neurons and postsynaptic reception of excitatory inputs, thereby exhibiting anti-epileptic action (88).

Zhu *et al.* explored methods of transplantation using human PSC (hPSC)-derived interneurons, which proved effective in both pilocarpine-induced temporal lobe epilepsy (TLE) and intrahippocampal kainate models.

Bershteyn *et al.* demonstrated that medial ganglionic eminence (MGE)-derived GABAergic interneurons from human ESCs (hESCs), delivered via single-dose intrahippocampal injection, markedly reduced seizures in medial temporal lobe epilepsy models, even achieving seizure-free outcomes and prolonged survival (26). Human iPSC (hiPSC)-derived MGE cells further attenuate progression from status epilepticus (SE) to chronic epilepsy. Notably, hiPSC-MGE transplantation during the late phase of SE reduces host interneuron loss and aberrant mossy fiber sprouting (MFS), while promoting physiological neurogenesis. Animals receiving hiPSC-MGE grafts post-SE exhibited improved cognitive and emotional function, potentially linked to the restoration of normal neuronal populations and suppression of pathological neurons (89–92).

Hattiangady *et al*. transplanted neural stem cells (NSCs) into animal models and observed that NSC-derived mature neurons migrated to the CA3 pyramidal cell layer or dentate granule cell layer, with approximately 17% differentiating into GABAergic interneurons. The percentage of normal hippocampal GABAergic neurons is about 10–15% (93), suggesting that transplanted cells can effectively replenish lost inhibitory circuits. Extrapolation of NeuN+ and GABA+ neuron counts per hippocampus revealed that NSC transplantation increased NeuN+ neurons by 21,280 and GABA+ interneurons by 19,040 per hippocampus. Neural stem cells differentiate into GABA^+^ inhibitory interneurons that replenish hippocampal neurons and significantly reduce seizure frequency (94). As NeuN is a marker for mature neurons, this quantitative evidence confirms that grafted NSCs successfully differentiated into functional neurons, directly replenishing neuronal losses induced by status epilepticus (SE). Critically, the specific restoration of inhibitory interneurons is essential, given that the loss of GABAergic interneurons constitutes a central mechanism underlying network hyperexcitability in epilepsy. The addition of 19,000 GABAergic interneurons effectively rebuilt the foundational units for inhibitory regulation within hippocampal circuitry.

Recent clinical trials by Neurona Therapeutics, focusing on inhibitory interneuron-based therapy, highlight the promise of NRTX-1001—a regenerative cell therapy derived from human PSCs (95). NRTX-1001 delivers long-term GABAergic inhibition to hyperexcitable neural networks via a single dose. Interim results show that 4 out of 5 low-dose recipients experienced >50% seizure reduction, with 3 being freed of debilitating focal seizures. Two subjects reported >95% seizure reduction at 16 and 21 months post-treatment. To date, NRTX-1001 has been well-tolerated in all participants (96), underscoring its potential as a groundbreaking clinical intervention for epilepsy.

### 3.2 Stem cells secrete neurotrophic factors

MSCs secrete diverse molecules via soluble factors and extracellular vesicles (EVs), including microvesicles (MVs), exosomes (EXs), and apoptotic bodies (97–99). EXs, which are critical mediators of intercellular communication, act through paracrine and endocrine pathways, delivering bioactive components such as chemokines, cytokines, interleukins, growth factors, proteins, free nucleic acids, and lipids (100, 101).

Waldau *et al.* demonstrated that medial ganglionic eminence-derived neural stem cells (MGE-NSCs) not only secrete bioactive factors but also differentiate into NeuN+ neurons (13%), S−100β + astrocytes (57%), and NG2 + oligodendrocyte progenitor cells (3%) in the post-epileptic hippocampus. The newly differentiated NeuN^+^ neurons may integrate into hippocampal neural circuitry reconstruction, likely contributing to the amelioration of epileptic symptoms. During epileptogenesis, astrocytic function may become impaired; however, graft-derived S-100β^+^ astrocytes (constituting a substantial proportion of differentiated cells) facilitate the restoration and maintenance of neural microenvironmental homeostasis. This attenuates excitotoxic damage while providing essential trophic support and neuroprotection to vulnerable neuronal populations. Additionally, oligodendrocyte progenitor cells can further mature into oligodendrocytes, which are critical for myelination and efficient neuronal impulse conduction. Given that myelination deficits are prevalent in the epileptic brain, these modest yet significant populations of graft-derived oligodendrocyte precursors may participate in remyelination and the functional recovery of neural transmission. Ten percent of these derivatives expressed *γ*-aminobutyric acid (GABA), while 50% expressed glial cell line-derived neurotrophic factor (GDNF). Replenishing depleted GABAergic inhibitory interneurons in the post-epileptic hippocampus directly restores inhibitory circuitry, thereby counteracting neuronal hyperexcitability and attenuating seizure activity. Furthermore, transplanted neural stem cells (NSCs) elevate glial cell line-derived neurotrophic factor (GDNF) expression in host astrocytes. This dual mechanism establishes a sustained reservoir of anti-epileptic factors that promotes neuronal survival/repair and maintains neural microenvironmental homeostasis, collectively mediating robust neuroprotective effects. Transplanted NSCs also restored GDNF levels in host astrocytes, suggesting that increased GDNF+ cells and astrocytic GDNF recovery underlie the therapeutic benefits of MGE-NSC grafts (102). Similarly, NSC-derived grafts exhibited variable expression of fibroblast growth factor-2 (FGF-2, 66%), insulin-like growth factor-1 (IGF-1, 59%), brain-derived neurotrophic factor (BDNF, 25%), and GDNF (45%) (94). The expression of neurotrophic factors within neural grafts underscores their contributory role in neuroprotection, neural regeneration, and tissue repair. Critically, distinct neurotrophic factors may engage in synergistic or complementary interactions during neural repair processes, collectively orchestrating the functional restoration of epileptically damaged neural tissue.

In a rat model of kainic acid (KA)-induced epilepsy, Wang *et al.* observed that transplanted adipose-derived stem cells (ADSCs) released BDNF, neurotrophin-3 (NT3), and neurotrophin-4 (NT4) in hippocampal tissues (32). These neurotrophic factors have broad anti-apoptotic effects (87), with BDNF modulating hippocampal long-term potentiation (LTP) and synaptic plasticity to enhance learning and memory (103). These restorative effects may be attributed to: Repair of hippocampal-prefrontal cortical circuitry, which critically regulates emotional processing. Modulation of neurotransmitter systems (e.g., serotonin [5-HT], dopamine). Rehabilitation of damaged limbic circuitry, particularly the hippocampus which governs both cognitive and affective functions. Thus, ADSC transplantation mitigates KA-induced hippocampal apoptosis and restores cognitive function. Additionally, Hattiangady *et al.* reported that NSC grafts express FGF-2 and vascular endothelial growth factor (VEGF), facilitating post-epileptic repair (104).

### 3.3 Stem cells attenuate epilepsy and complications via antioxidant and anti-inflammatory effects

Liu *et al.* found that olfactory mucosa-derived MSCs (OM-MSCs) reduced post-epileptic neuroinflammation by suppressing astrogliosis and microgliosis, alleviating demyelination and neuronal loss. OM-MSC-treated groups exhibited lower pro-inflammatory cytokines (Tnf, Il1b, and Il6) and elevated anti-inflammatory IL-10, promoting neural repair. OM-MSCs also recruited regulatory T cells (Tregs) to suppress inflammation and enhance microglial repair activity, ultimately restoring cognitive function (105, 106). The therapeutic benefits of MSCs in inflammatory brain disorders may arise from the paracrine actions of EVs and soluble factors (107, 108). Notably, MSC-derived EVs exhibit potent anti-inflammatory properties post-brain injury (109–111), capitalizing on their low immunogenicity, immunomodulatory capacity, and multipotency (112).

Xian *et al.* demonstrated that MSC-derived EXs (MSC-Exo) attenuated lipopolysaccharide (LPS)-induced cytotoxicity, reactive astrogliosis, and inflammatory cytokine secretion (TNF-*α* and IL-1β) *in vitro* (113). Luo *et al.* highlighted the antioxidant activity of MSC-EVs, which mitigated oxidative stress, reversed hippocampal neuronal morphological changes, and restored mitochondrial dysfunction post-seizures, improving cognitive outcomes (17). Long *et al.* reported that intranasal delivery of human bone marrow MSC-derived A1-EXs post-SE suppressed pro-inflammatory cytokines (TNF-*α*, IL-1β, MCP-1, SCF, MIP-1α, GM-CSF, and IL-12) while elevating anti-inflammatory factors, reducing microglial activation, and attenuating the inflammatory cascade (114). Shoja *et al.* demonstrated that dental pulp stem cells (DPSCs) reduced astrocyte proliferation and complexity in the hippocampal CA1 region, alleviating post-epileptic inflammation and cell death (62).

### 3.4 Stem cell grafts protect neuronal survival and suppress aberrant neurogenesis

Dentate gyrus neurogenesis post-SE follows a biphasic pattern: excessive aberrant neurogenesis occurs during the acute/subacute phases (115, 116), while diminished neurogenesis occurs in the chronic phase (91). Hattiangady *et al.* showed that early NSC transplantation in SE models significantly reduced aberrant neurogenesis and protected neuronal survival. Compared to controls, SE alone reduced normal neurons by 87%, whereas SE + NSC grafts limited loss to 47%. NSC grafts also suppressed MFS, a hallmark of hippocampal hyperexcitability (94).

Huang *et al.* observed that human umbilical cord MSC (HUMSC) transplantation preserved hippocampal pyramidal neuron integrity and inhibitory circuits in chronic epilepsy, mitigating pilocarpine-induced neuronal loss in CA1 and CA3. Timm staining revealed excessive MFS into the inner molecular layer (IML) and CA3 in SE rats, which HUMSC grafts significantly attenuated (117, 118). Fukumura *et al.* confirmed that MSC infusion increased GABAergic interneurons and NeuN+ neurons in SE hippocampi, while suppressing MFS according to Timm staining (119). Stem Cell Therapy Mitigates SE-Induced Aberrant Neurogenesis by Suppressing Ectopic Migration and Modulating Overall Neurogenic Dynamics: 1. Suppression of Ectopic Migration: Neural stem cell (NSC) transplantation into SE models significantly reduces mossy fiber sprouting (MFS) area by 58%, indirectly indicating inhibition of newborn neuron ectopic migration (120, 121). This effect is mediated through: Secretion of neurotrophic factors (e.g., BDNF, FGF-2) and extracellular matrix components (e.g., integrins) that guide neuronal migration along physiological paths to the dentate granule layer, minimizing aberrant colonization of the hilus and CA3 (122). Synaptic anchoring of ectopic neurons by NSC-derived mature neurons, restricting abnormal projections via functional connectivity (123). 2. Modulation of Aberrant Neurogenesis NSC transplantation reduces pathological neurogenesis by 73% while restoring chronic-phase neurogenesis to physiological levels (124). Key mechanisms include: 1. Promotion of neuronal maturation: BDNF released by ADSCs accelerates dendritic development and synaptic integration of newborn neurons, reducing their epileptogenicity (125, 126). 2. Neurogenic cycle rebalancing: MSC-derived extracellular vesicles (MSC-EVs) restore mitochondrial function in neural stem cells, normalizing the proliferation-differentiation equilibrium to prevent chronic-phase neurogenic suppression (17, 127, 128).

Collectively, preclinical evidence supports stem cell therapy in counteracting epileptogenic neurogenesis (Table 1).

**Table 1:** Selected basic studies on stem cell therapy for epilepsy.

Authors	Stem cell variety	Method of transplantation	Type of experiment and timing of treatment	Research results
Zhu et al. 2023 (159)	Human induced pluripotent stem cell-derived GABAergic interneurons	Intra-hippocampal injection	Basic experiment: (Model: Nod Scid Gamma (NSG) mouse temporal lobe epilepsy model); Early transplantation of GABAergic interneurons (about 3 weeks).	Transplanted neurons modulate host inhibition without over-inhibition, ameliorate seizures, and restore neuropsychiatric comorbidity after epilepsy induction in mouse models (159). No seizures within 9 months; 78% synaptic integration of grafted cells without excessive inhibition.
Bershteyn et al. 2023 (26)	(MGE) Medullary origin GABAergic interneurons	Intra-hippocampal injection	Basic experiment: (model: NOG mouse temporal lobe epilepsy model); Early transplantation of MGE (about 4–5 weeks)	The ability of neurons to persist and integrate functionally into wild-type and epileptic rodents significantly suppresses seizures and improves spatial memory as well as survival in mouse models (26). Complete disappearance of seizure and prolonged survival after a single transplantation; 2.3-fold increase in GABA release compared with controls.
Hattiangady et al. 2020 (94)	Neural stem cell	Intra-hippocampal injection	Basic experiment: (model: Fischer 344 rat epilepsy model); Early transplantation of Neural stem cell (about 1 weeks)	Reduce abnormal neurogenesis, suppress seizures, enhance memory and survival, and alleviate depressive behaviors in chronic epilepsy models. (94).
Wang et al. 2021 (32)	Adipose mesenchymal stem cells	Intra-hippocampal injection	Basic experiment: (model: SD rat TLE model); Not specified	Transplantation reduced the number of seizures in rats and was able to release neuroprotective factors such as BDNF, NT3 and NT4, thereby inhibiting KA-induced apoptosis in rat hippocampal tissue (32). Secretion of BDNF, NT3, NT4; 47% reduction in hippocampal apoptotic cells, 35% improvement in learning memory scores.
Xian et al. 2019 (113)	Extracellular vesicles of umbilical cord mesenchymal stem cells	Intracerebroventricular injection	Basic experiment: (Model: C57B/6 male mouse epilepsy model); Not specified	Transplantation reduces hippocampal reactive astrocyte proliferation, attenuates SE-induced hippocampal inflammatory responses, and restores SE-induced learning and memory deficits in a mouse model of SE (113).
Luo et al. 2021 (17)	Extracellular vesicles of umbilical cord mesenchymal stem cells	Tail vein injection	Basic experiment: (Model: C57BL/6 mouse epilepsy model); Early transplantation of Extracellular vesicles of umbilical cord mesenchymal stem cells (about 2 h)	Restore seizure-induced neuronal morphological alterations and hippocampal mitochondrial dysfunction, and ameliorate hippocampal neuronal damage-related sequelae of seizures in the mouse hippocampus (17).
Shoja et al. 2024 (62)	Dental pulp mesenchymal stem cells	Intra-hippocampal injection	Basic experiment (model: Sprague–Dawley rat epilepsy model); Early transplantation of Dental pulp mesenchymal stem cells (about 1 month)	Transplantation improves memory function in a rat seizure model and reduces hippocampal neuronal apoptosis and gliosis after seizure onset (62).
Liu et al. 2023 (105)	Olfactory mucosa MSCs (OM-MSCs)	Intra-hippocampal injection	Basic experiment: (Model: C57BL/6 mouse epilepsy model); Early transplantation of OM-MSCs (About 4–44 days)	IL-1β, TNF-α decreased by 40–50%, IL-10 increased 2.1-fold; 60% increase in microglia repair phenotype. Evidence that MSCs can remodel the inflammatory microenvironment of the epileptic brain through immunomodulation (105).
Fukumura et al. 2018 (119)	MSCs	Tail vein injection	Basic experiment: (Model: Adult male Sprague–Dawley rats); Early transplantation of MSCs (About 1 days)	GABAergic neurons increased by 32 per cent and MFS scores decreased by 45 percent. Synergistic effects of associative inhibitory neuronal replenishment and structural remodeling (119).

### 3.5 Stem cell therapy ameliorates enzyme deficiency-induced epilepsy

Rybova *et al.* demonstrated that hematopoietic stem cell transplantation prevented disease progression and spinal demyelination in a mouse model of spinal muscular atrophy with progressive myoclonic epilepsy (SMA-PME) caused by acid ceramidase (ACDase) deficiency, improving behavioral outcomes (85). However, the underlying mechanisms warrant further exploration.

Emerging evidence from completed and ongoing clinical trials supports the therapeutic efficacy of stem cell-based interventions in epilepsy, as summarized in Tables 2, 3.

**Table 2:** Selected clinical studies on stem cell therapy for epilepsy.

Clinical trial serial number	Cell type	Disease type	Method of transplantation	Sample size	Results
NCT02497443	BMSCs	Refractory symptomatic epilepsy	Intravenous + intrathecal injection	*n* = 22	In the cell therapy group, intravenous/intrathecal administration caused no severe adverse events. Of the recipients, 5/10 had cognitive improvement, 6/10 had reduced anxiety, and 2/10 had alleviated depression (depression worsened in 1) (160).
NCT00916266	BMMCs	Refractory medial temporal lobe epilepsy	Selective intra-arterial infusion	*n* = 20	At the 6-month follow-up, 40% of patients were completely freed of seizures, 25% exhibited a 70–99% reduction in seizure frequency, 15% exhibited a 50–69% reduction, and 20% exhibited less than 50% reduction in seizure frequency (161).
NCT03676569	ADRCs	Refractory epilepsy due to autoimmune mechanisms	Intrathecal injection	*n* = 6	At 6-month follow-up, 1 patient was completely freed of seizures and 2 had 50% seizure reduction. Transient clinical improvement occurred post-first ADRC infusion in 1 patient; another showed temporary EEG improvement (3–4 months) (162).
ChiCTR2200055357	OM-MSCs	Chronic refractory epilepsy	Intrathecal injection	*n* = 1	The treatment relieved the patient’s cerebral atrophy, significantly alleviated epileptic symptoms (generalized tonic clonic seizures disappeared, and sudden collapse symptoms were significantly reduced) (105).
Unregistered	BMNCs+BMMSCs	Drug-resistant epilepsy	Intravenous + intrathecal injection	*n* = 19	In the trial, 15/19 patients (78.9%) reported mild to moderate adverse events. Six patients had significant seizure reduction (from 56 to 1–14 per week). Two displayed altered seizure patterns, shorter durations, and faster recovery. Five patients were freed of status epilepticus (previously requiring hospitalization) and reduced/discontinued medications (163).
NCT02497443	BMSCs	Refractory symptomatic epilepsy	Intravenous + intrathecal injection	*n* = 67	Cell therapy had a significant effect on the number of seizures in patients in the MSC group, as evinced by a reduction in the number of combined seizures (164).

BMSCs, bone marrow-derived mesenchymal stem cells; BMMCs, bone marrow-derived mononuclear cells; ADRCs adipose-derived regenerative cells; OM-MSCs, olfactory mucosal mesenchymal stem cells; BMNCs, bone marrow-derived nucleated cells; BMMSCs, bone marrow-derived mesenchymal stem cells.

**Table 3:** Ongoing clinical trials on stem cell therapy for epilepsy.

NCT serial number	Cell type	Epilepsy type	Sample size	Results
NCT05135091	NRTX-1001	Drug-resistant focal epilepsy in adults	*n* = 40	Among 5 subjects receiving lower-dose NRTX-1001 therapy, 4 demonstrated >50% reduction in seizure frequency, with 3 being freed of their most disabling focal seizure type. Two subjects exhibited sustained >95% seizure reduction from the baseline, persisting for 16 and 21 months post-NRTX-1001 administration, respectively.
NCT05886205	iPSC-EXOs	Refractory focal epilepsy	*n* = 34	NA
NCT06280092	ADSCs	Epilepsy	*n* = 5	NA
NCT06638970	UCMSC secretome	Drug-refractory epilepsy	*n* = 54	NA
NCT03676569	ADSCs	Autoimmune refractory epilepsy	*n* = 6	NA
NCT00916266	BMSCs	Temporal lobe Epilepsy	*n* = 20	NA
NCT02497443	MSCs	Epilepsy	*n* = 67	NA

NRTX-1001, human pluripotent stem cell-derived interneurons; iPSC-EXOs, induced pluripotent stem cell exosomes; ADSCs, adipose mesenchymal stem cells; UCMSC secretome, umbilical cord mesenchymal stem cell exosomes; BMSCs, bone marrow mesenchymal stem cells; MSCs, mesenchymal stem cells.

## 4 Discussion

Stem cell therapy holds immense potential for epilepsy treatment; however, significant challenges remain, including ensuring safety, enhancing efficacy, overcoming technical bottlenecks, and addressing ethical concerns. Future research must focus on optimizing methodologies and conducting large-scale, long-term clinical trials to advance stem cell therapy toward clinical use, ultimately delivering safe, effective, and sustainable treatments for patients.

### 4.1 Current challenges

#### 4.1.1 Sources of and selection of stem cells

Stem cells are derived from diverse sources, including ESCs, adult stem cells, and iPSCs, each possessing distinct advantages and limitations that necessitate careful selection for optimal therapeutic outcomes. Currently, MSCs have gained prominence in clinical use due to their multifaceted therapeutic properties. These include superior regenerative potential, antioxidant stress mitigation, and anti-apoptotic capabilities, which collectively contribute to their critical role in disease intervention. Moreover, MSCs exhibit remarkable secretory functions through the production of bioactive cytokines and EVs, facilitating intercellular communication and tissue repair. The relative ease of isolation from accessible tissues (e.g., adipose tissue, bone marrow, and the umbilical cord) combined with minimal ethical concerns has significantly facilitated their widespread clinical utilization (112–114). While other stem cell types demonstrate unique therapeutic potentials, their full clinical implementation requires resolution of ethical controversies and optimization of accessibility. Future advances in overcoming these challenges may unlock substantial therapeutic prospects across various stem cell platforms, enabling personalized regenerative strategies tailored to specific pathological contexts (129–131). Other stem cell types, while promising, require the addressing of ethical concerns and the overcoming of accessibility barriers to unlock their full potential.

#### 4.1.2 Post-transplantation survival and function

Ensuring stem cell survival and functional integration into host neural networks post-transplantation remains a major hurdle. Key factors requiring further investigation include transplantation targets, mechanisms of action, cell types, and therapeutic outcomes (132). Key obstacles include inflammatory mediators (e.g., IL-1β, TNF-*α*), oxidative stress, and synaptic mismatch, which collectively impair graft viability and neural circuit integration (133). Enhancing cell survival requires strategies such as preconditioning stem cells with antioxidants (e.g., N-acetylcysteine) or genetic modifications to overexpress anti-apoptotic proteins (e.g., BCL-2). Concurrently, promoting synaptic connectivity between donor-derived neurons and host networks necessitates precise delivery timing and microenvironmental optimization, including neurotrophic support (BDNF, GDNF) and activity-dependent plasticity modulation. Critical research priorities are as follows:

Transplantation protocol optimization: Determining ideal cell types (e.g., GABAergic progenitors vs. MSCs), doses, and delivery routes (intrahippocampal vs. intravenous). Microenvironmental modulation: Utilizing biomaterials like injectable hydrogels to sustain neuroprotective factor release and shield grafts from inflammatory damage. Mechanistic clarification: Distinguishing therapeutic effects from direct synaptic integration versus paracrine signaling using optogenetic silencing and lineage-tracing technologies. Emerging tissue engineering approaches, such as 3D-printed scaffolds with topographical cues, show promise for guiding axonal growth and improving graft-host connectivity (134). Standardized metrics for evaluating therapeutic efficacy—including a reduction in seizure frequency, cognitive recovery, and functional MRI-based graft tracking—are essential for clinical translation. Future advances will require interdisciplinary efforts to address immune compatibility, long-term safety, and scalability, ultimately positioning stem cell therapy as a viable option for drug-resistant epilepsy (135).

#### 4.1.3 Long-term efficacy and safety

The long-term safety profile and sustained therapeutic effects of stem cell transplantation remain incompletely characterized, necessitating rigorous longitudinal investigations to address critical knowledge gaps. Key unresolved concerns include: (1) immune-mediated rejection of allogeneic grafts, particularly in non-immunoprivileged brain regions; (2) tumorigenic potential associated with residual undifferentiated pluripotent cells; and (3) infection risks from xenogenic components in culture systems. Moreover, heterogeneity in cell sourcing (autologous vs. allogeneic) and preparation protocols (enzymatic dissociation vs. mechanical isolation) may introduce variability in clinical outcomes and safety profiles (136). Current evidence from preclinical models demonstrates sustained seizure suppression (>6 months post-transplantation) through GABAergic interneuron integration and neurotrophic modulation. However, clinical translation requires systematic evaluation of the following.

Chronic neuroinflammatory responses: Monitoring microglial activation (via TSPO-PET imaging) and cytokine profiles (IL-6, TGF-β1).
Oncogenic surveillance: Implementing multimodal screening protocols combining liquid biopsy (circulating tumor DNA) and annual MRI for tumor detection.

#### 4.1.4 Standardization of stem cell processing

The clinical translation of stem cell therapies for epilepsy remains hindered by a critical lack of standardized protocols spanning cell selection, processing, expansion, and transplantation (132). Variability in cell sources (e.g., autologous vs. allogeneic), culture conditions, and methods of differentiation compromises both reproducibility and therapeutic reliability (137). Establishing unified industry standards is imperative to ensuring consistency in cell viability (>90%), purity (<1% undifferentiated cells), and functional potency across studies. Advances in automated, closed-system bioreactors enable scalable production of clinical-grade stem cells while minimizing contamination risks. Integration of chemically defined culture matrices and AI-driven monitoring systems further enhances batch-to-batch consistency. Collaborative efforts between regulatory bodies and scientific consortia are essential to harmonizing epilepsy-specific benchmarks, including synaptic integration capacity and subtype-specific differentiation ratios, thereby facilitating multicenter trial comparability and accelerating therapeutic validation.

#### 4.1.5 Ethical and legal considerations

Stem cell advances raise ethical, moral, and legal challenges, particularly regarding human embryo use, therapeutic cloning, and tissue sourcing. Informed consent for tissue procurement is essential to mitigating ethical disputes. Ethical debates often center on treatment risks, side effects, safety, and societal impact (138). Clinical trials must adhere to internationally recognized ethical standards, be scientifically rigorous, and adhere to principles of patient protection. These issues demand thorough consideration during research and clinical translation.

### 4.2 Future research directions

#### 4.2.1 Basic research

The use of stem cells in epilepsy treatment hinges on elucidating their capacity to modulate hyperexcitable neuronal networks and repair epileptogenic circuitry (139). Focal epilepsies, and particularly temporal lobe epilepsy (TLE), are characterized by hippocampal neuron loss, gliosis, and aberrant synaptic reorganization—pathological features that may be ameliorated through stem cell-derived interventions (140). Preclinical studies have highlighted the potential of MSCs to suppress neuroinflammation via anti-apoptotic and immunomodulatory cytokine secretion (e.g., TGF-*β* and IL-10), while iPSC-derived GABAergic interneurons may restore inhibitory tone in seizure-prone regions such as the dentate gyrus. However, challenges persist in ensuring long-term survival, targeted migration, and functional synaptic integration of transplanted cells within sclerotic hippocampi. Recent advances in optogenetic control of grafted cells and single-cell transcriptomic profiling of host-graft interactions offer novel tools to elucidate mechanisms of circuit repair (141). Further research must prioritize optimizing methods of delivery (e.g., biomaterial scaffolds for localized grafting) and mitigating risks such as ectopic cell proliferation or unintended network dysregulation (142). Further understanding of epigenetic and metabolic crosstalk between stem cells and the epileptic microenvironment will be critical to translating these therapies into clinically viable, precision-based interventions.

#### 4.2.2 Clinical trials

Large-scale, multicenter, RCTs need to be conducted to rigorously evaluate the efficacy and safety of stem cell therapy in epilepsy. Stem cell therapies are increasingly being tested in clinical trials for various conditions, such as diabetes (143–145) and neurodegenerative disorders (e.g., Parkinson’s disease, Alzheimer’s disease, and multiple sclerosis) (146–148). However, challenges persist in standardizing cell types (e.g., MSCs vs. iPSC-derived inhibitory interneurons), delivery routes (intravenous vs. intracerebral), and dosing protocols across diverse cohorts. Recent advances in epilepsy research have prompted multiple international trials assessing stem cell safety and efficacy, with preliminary results showing promise. Increasing multicenter and multinational collaboration will enhance sample diversity and enable cross-regional comparisons of therapeutic outcomes. A critical gap remains in validating objective efficacy endpoints beyond seizure diaries, such as electrophysiological biomarkers or functional neuroimaging correlates of network stabilization. Concurrently, regulatory agencies must balance accelerated approval pathways for urgent unmet needs with robust post-marketing surveillance to monitor risks like graft overgrowth or autoimmune reactions. By integrating patient-reported outcomes with mechanistic biomarkers, future trials can bridge the translational gap while maintaining ethical and scientific rigor.

#### 4.2.3 Personalized treatment

In the context of epilepsy, stem cell therapy has emerged as a promising avenue for personalized treatment. Given the highly heterogeneous nature of epilepsy, with diverse etiologies and clinical manifestations among patients, a one-size-fits-all approach is often ineffective. Precision biomarkers, genetic profiling, and advanced neuroimaging can guide patient stratification, real-time monitoring of transplanted cells, and evaluation of therapeutic responses. Tailoring treatments to individual biological, genetic, and clinical profiles remains a key challenge and opportunity for improving outcomes (149). Precision biomarkers, such as seizure-associated miRNA profiles or PET/MRI-based metabolic signatures, could further stratify patients for autologous vs. allogeneic cell therapies and track graft viability post-transplantation (150). Genetic profiling is another essential tool. Epilepsy is known to have a significant genetic component, and different genetic mutations can lead to distinct pathophysiological mechanisms. Through comprehensive genetic profiling, researchers can determine the genetic background of a patient’s epilepsy. This knowledge can then be used to engineer stem cells that are genetically modified to correct the underlying genetic defects. However, tailoring stem cell treatments to individual biological, genetic, and clinical profiles is not without challenges. The complexity of the epileptic brain, with its multiple interacting neural networks and compensatory mechanisms, hampers the precise prediction of how the transplanted stem cells will behave.

#### 4.2.4 Technological innovations

In the pursuit of effective stem cell therapies for epilepsy, emerging technologies are playing a transformative role. CRISPR-based gene editing has emerged as a powerful tool with immense potential. Epilepsy is often associated with various genetic mutations, and CRISPR technology allows for the precise targeting and correction of these epilepsy-associated genetic defects (151). By modifying the genetic code within stem cells, researchers can potentially create cells that are resistant to abnormal neural firing patterns characteristic of epilepsy (152). If, for example, a specific gene mutation is causing an over-excitability of neurons in the epileptic brain, CRISPR can be used to edit the gene to restore normal neuronal function. This not only holds promise for treating the underlying cause of epilepsy but also for enhancing the therapeutic properties of stem cells, making them more effective in suppressing epileptic seizures. Single-cell genetic reprogramming techniques are also revolutionizing the field. These techniques enable scientists to exercise precise control over stem cell differentiation and function. In the context of epilepsy treatment, this means the ability to generate specific types of neural cells that can integrate seamlessly into the epileptic brain and restore normal neural circuitry (153). For instance, precisely reprogramming stem cells at the single-cell level enables the creation of inhibitory neurons, which are often deficient in epileptic brains. These inhibitory neurons can then be transplanted into the epileptic focus to counterbalance the excessive excitatory activity, potentially halting seizure propagation. This targeted approach, made possible by single-cell genetic reprogramming, offers a novel cell source for epilepsy treatment that was previously unattainable. Advances in cell processing and methods of transplantation are equally crucial. Biomaterial scaffolds, for example, can provide a supportive framework for transplanted stem cells. In the epileptic brain, which has a complex and often damaged neural environment, these scaffolds can help guide the migration and integration of stem cells to the appropriate regions (154). They can also mimic the natural extracellular matrix, promoting cell survival and differentiation. Combining these innovative cell processing and transplantation techniques will enable the safety and effectiveness of stem cell therapies for epilepsy to be significantly enhanced, bringing us closer to a viable treatment option for this challenging neurological disorder.

#### 4.2.5 Combination therapies

Combining stem cell therapy with existing treatments—such as AEDs, the ketogenic diet (KD), or neuromodulation—may yield synergistic benefits. AEDs are currently the cornerstone of epilepsy treatment, aiming to control seizure frequency and severity (155). However, a significant proportion of patients remain refractory to these medications. Stem cell therapy, with its potential to modulate the epileptic brain at a cellular and molecular level, could complement AEDs. The KD, a high-fat, low-carbohydrate regimen for drug-resistant epilepsy, modulates biochemical pathways to reduce seizures (156). The KD increases the production of ketone bodies, which can act as an alternative energy source for the brain. In the context of epilepsy, this metabolic shift may also have a direct impact on neuronal excitability. Investigating its synergy with stem cell therapy could enhance efficacy. Stem cells, when transplanted, might respond to the altered metabolic environment induced by the KD. For instance, the increased availability of ketone bodies could potentially facilitate the survival and differentiation of transplanted stem cells into functional neural cells, thereby enhancing their overall anti-epileptic effect (157). Investigating its synergy with stem cell therapy could enhance efficacy. Similarly, integrating stem cell grafts with deep brain stimulation (DBS) targeting the anterior nucleus of the thalamus (ANT) may offer novel strategies for drug-refractory epilepsy (158). This multi-modal approach holds great promise for improving treatment outcomes for patients with drug-refractory epilepsy.

#### 4.2.6 Ethical and legal frameworks

Establishing robust ethical and legal guidelines is essential to ensuring compliance and protecting patient rights. Transparent reporting of clinical trial data, public disclosure of research protocols, and rigorous oversight will foster trust and standardization. As regulations evolve, stem cell research and use of those cells in epilepsy must align with international ethical standards, balancing innovation with societal and moral considerations.

## 5 Conclusion

Stem cell therapy for epilepsy, as an emerging therapeutic strategy, has demonstrated significant potential and promise in preclinical studies and early-phase clinical trials. It offers multifaceted mechanisms—including differentiation into interneurons, antioxidant stress mitigation, secretion of cytokines and EVs, neuroprotection, and immunomodulation—to alleviate clinical symptoms, partially restore neural function, reduce seizure frequency, and delay disease progression. However, addressing existing challenges, such as optimizing safety and efficacy, remains critical to ensuring successful clinical translation. Future research will further our understanding of stem cell biology and accelerate their widespread use in epilepsy treatment. Innovations in this field will prioritize precision-based, personalized, and safe approaches, with interdisciplinary collaboration and technological advances serving as key drivers. As research and technology evolve, stem cell therapy may provide more effective and safer treatment options for patients with epilepsy. With progress in regenerative medicine, stem cell-based approaches hold promise as viable therapies for diverse neurological disorders, ultimately improving the quality of life of patients and their families.
